# Tetrakis(μ_3_-9-oxa-10-boraanthracen-10-olato)tetrakis[(diethyl ether)lithium]

**DOI:** 10.1107/S1600536811041201

**Published:** 2011-10-12

**Authors:** Daniel Franz, Hans-Wolfram Lerner, Dominik Büttner, Michael Bolte

**Affiliations:** a Institut für Anorganische Chemie, J. W. Goethe-Universität Frankfurt, Max-von-Laue-Strasse 7, 60438 Frankfurt/Main, Germany

## Abstract

The title compound, [Li_4_O_4_(C_12_H_8_BO)_4_(C_4_H_10_O)_4_], features a Li_4_O_4_ cube. Each Li atom in the cube is additionally coordinated by a diethyl ether mol­ecule and each O atom in the cube carries a 9-oxa-10-boraanthracene residue. The crystal studied was a non-merohedral twin [twin law (-1 0 0 / 0 0 1 / 0 1 0); the contribution of the major twin component refined to 0.553 (3)] emulating apparent tetra­gonal symmetry, whereas the actual crystal system is just ortho­rhom­bic.

## Related literature

For chalcogenolate ligands used to stabilize transition metal centers, see: Wolczanski (2009 ▶); Kückmann *et al.* (2005 ▶, 2008 ▶, 2010 ▶). For synthetic details, see: Davidson & French (1960 ▶); Mikoshiba *et al.* (2003 ▶); Zuideveld *et al.* (2002 ▶); Knizek & Nöth (2000 ▶). For related structures, see: Kückmann *et al.* (2007 ▶); Lerner *et al.* (2002 ▶).
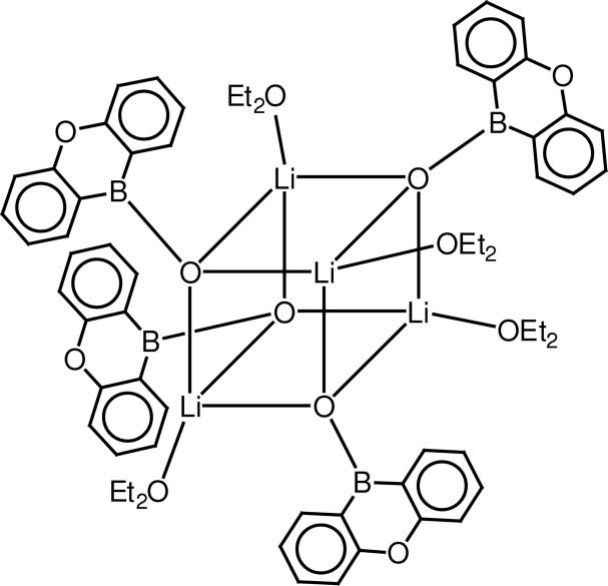


## Experimental

### 

#### Crystal data

[Li_4_O_4_(C_12_H_8_BO)_4_(C_4_H_10_O)_4_]
*M* *_r_* = 1104.22
Orthorhombic, 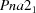

*a* = 21.4805 (8) Å
*b* = 17.6053 (10) Å
*c* = 17.5723 (8) Å
*V* = 6645.3 (5) Å^3^
*Z* = 4
Mo *K*α radiation
μ = 0.07 mm^−1^
*T* = 173 K
0.30 × 0.30 × 0.25 mm

#### Data collection

Stoe IPDS II two-circle diffractometer
77138 measured reflections
6262 independent reflections
4842 reflections with *I* > 2σ(*I*)
*R*_int_ = 0.114

#### Refinement

*R*[*F* ^2^ > 2σ(*F* ^2^)] = 0.077
*wR*(*F* ^2^) = 0.204
*S* = 1.04
6262 reflections
758 parameters
29 restraints
H-atom parameters constrained
Δρ_max_ = 1.10 e Å^−3^
Δρ_min_ = −0.35 e Å^−3^

### 

Data collection: *X-AREA* (Stoe & Cie, 2001 ▶); cell refinement: *X-AREA*; data reduction: *X-AREA*; program(s) used to solve structure: *SHELXS97* (Sheldrick, 2008 ▶); program(s) used to refine structure: *SHELXL97* (Sheldrick, 2008 ▶); molecular graphics: *XP* (Sheldrick, 2008 ▶); software used to prepare material for publication: *SHELXL97* and *PLATON* (Spek, 2009 ▶).

## Supplementary Material

Crystal structure: contains datablock(s) I, global. DOI: 10.1107/S1600536811041201/bg2423sup1.cif

Structure factors: contains datablock(s) I. DOI: 10.1107/S1600536811041201/bg2423Isup2.hkl

Additional supplementary materials:  crystallographic information; 3D view; checkCIF report

## Figures and Tables

**Fig. 1.**
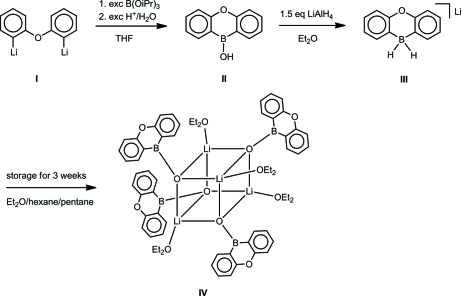
Synthesis of the title compound.

**Fig. 2.**
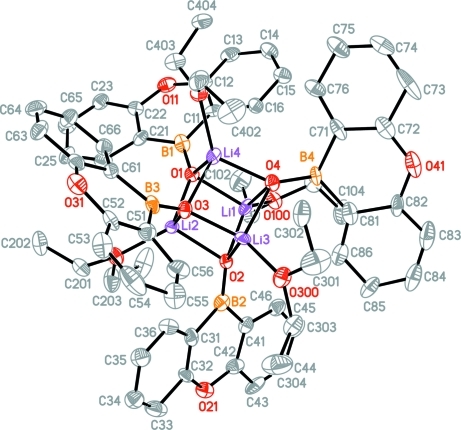
Perspective view of the title compound with displacement ellipsoids drawn at the 30% probability level. H atoms omitted for clarity.

## supplementary crystallographic information

### Comment

In this study, the accordance of the structural characteristics between the alkali metal salts of borinic acids, M^+^[EBR_2_]^-^ (E = O), and the related silyl chalcogenolates M^+^[ESiR_3_]^-^ (E = O, S, Se, Te) is taken into account. It has been shown that chalcogenolate ligands of the type [ESiR_3_]^-^ (E = O, S, Se, Te) were used to stabilize transition metal centers (Wolczanski, 2009; Kückmann *et al.*, 2005, 2008, and 2010). These ligands have also been applied in the field of macromolecular chemistry. Normally, transition metal complexes possess 6 e^-^ chalcogenolate ligands in a *µ*_3_ binding mode. Recently, however, we have shown that the anion of the mixed valence Mn(I/II) complex [Na(thf)_6_][(OC)_3_Mn(*µ*-SSi*t*Bu_3_)_3_MnSSi*t*Bu_3_] contains a terminal thiolate ligand with a linear Mn—S—Si unit (Kückmann *et al.*, 2008). The prerequisite for electronic communication between the metal center and the ligand is thus fulfilled. By an attempt to synthesize compound III (Fig. 1) we obtained as a by-product single crystals of the title compound (IV). The starting materials I and II were synthesized by modification of the procedures reported in the literature (Mikoshiba *et al.*, 2003; Davidson & French, 1960; Zuideveld *et al.*, 2002). IV displays a heterocubane structure in the solid state. Related structural motifs are found in the silyl chalcogenolates (Na[OSi*t*Bu_3_])_4_, (Na[OSiPh*t*Bu_2_])_4_, and ([Na(thf)][OSiMePh_2_])_4_ (Lerner *et al.* 2002; Kückmann *et al.*, 2007). In conclusion, the solid-state structure of IV provides further evidence for the diagonal relationship between B and Si.

The title compound (Fig. 2) features a Li_4_O_4_ cube. Each Li atom in the cube is additionally coordinated by a diethyl ether molecule and each O atom in the cube carries a 9-oxa-10-bora-anthryl residue.

### Experimental

All transformations were carried out under an atmosphere of dry nitrogen using standard Schlenk techniques and carefully dried solvents. The ^11^B NMR spectrum was recorded on a Bruker Avance 300. The borinic acid II (1.18 g, 6.0 mmol) was dissolved in Et_2_O (40 ml) and cooled to 0 °C. Under vigorous stirring a solution of Li[AlH_4_]  (9 mmol) in Et_2_O (19 mL) was added dropwise over a period of 30 min (beforehand, few small crystallites of 2,2'-biquinoline had been added to the ethereal Li[AlH_4_] solution as a color indicator for the consumption of Li[AlH_4_]). The mixture was allowed to warm to room temperature over night yielding a pale yellow suspension. In the ^11^B NMR spectrum (in C_6_D_6_/THF (1:1), referenced to external BF_3_^.^Et_2_O) a triplet was observed at -24.0 ppm (^1^*J*_BH_ = 78 Hz) which indicated conversion into the putative 9,9-dihydrido-9-oxa-10-borataanthracene (III) (*cf.* lithium 9,9-dihydrido-9-boratafluorene: *δ*(^11^B) = -22.3 (^1^*J*_-H_ = 77 Hz) in CDCl_3_) (Knizek & Nöth, 2000). The solid was removed by filtration. The clear solution was subject to a crystallization experiment implementing gas phase diffusion exchange of solvent with a reservoir containing a mixture of hexane/pentane (15 ml/15 ml). After three weeks a few cubic crystals of the title compound (IV) were obtained.

### Refinement

Due to the absence of anomalous scatterers, the absolute structure could not be determined and 6701 Friedel pairs were merged. All H atoms were geometrically positioned and refined using a riding model with fixed individual displacement parameters [U(H) = 1.2 *U*_eq_(C) or U(H) = 1.5 *U*_eq_(C_methyl_)] using a riding model with C—H ranging from 0.95 Å to 0.99 Å. The C—C distances in the ether molecules were restrained to be equal within an effective e.s.d. of 0.01 Å. The crystal was a non-merohedral twin emulating apparent tetragonal symmetry. The *b* and *c* axis are of very similar lengths. The twin law is (-1 0 0 / 0 0 1 / 0 1 0) and the contribution of the major twin component refined to 0.553 (3).

### Crystal data

**Table:**

[Li_4_O_4_(C_12_H_8_BO)_4_(C_4_H_10_O)_4_]	*F*(000) = 2336
*M**_r_* = 1104.22	*D*_x_ = 1.104 Mg m^−^^3^
Orthorhombic, *P**n**a*2_1_	Mo *K*α radiation, λ = 0.71073 Å
Hall symbol: P 2c -2n	Cell parameters from 31407 reflections
*a* = 21.4805 (8) Å	θ = 2.6–27.6°
*b* = 17.6053 (10) Å	µ = 0.07 mm^−^^1^
*c* = 17.5723 (8) Å	*T* = 173 K
*V* = 6645.3 (5) Å^3^	Block, colourless
*Z* = 4	0.30 × 0.30 × 0.25 mm

### Data collection

**Table:**

Stoe IPDS II two-circle diffractometer	4842 reflections with *I* > 2σ(*I*)
Radiation source: fine-focus sealed tube	*R*_int_ = 0.114
graphite	θ_max_ = 25.0°, θ_min_ = 2.6°
ω scans	*h* = −25→24
77138 measured reflections	*k* = −20→20
6262 independent reflections	*l* = −20→20

### Refinement

**Table:**

Refinement on *F*^2^	Primary atom site location: structure-invariant direct methods
Least-squares matrix: full	Secondary atom site location: difference Fourier map
*R*[*F*^2^ > 2σ(*F*^2^)] = 0.077	Hydrogen site location: inferred from neighbouring sites
*wR*(*F*^2^) = 0.204	H-atom parameters constrained
*S* = 1.04	*w* = 1/[σ^2^(*F*_o_^2^) + (0.1379*P*)^2^] where *P* = (*F*_o_^2^ + 2*F*_c_^2^)/3
6262 reflections	(Δ/σ)_max_ < 0.001
758 parameters	Δρ_max_ = 1.10 e Å^−^^3^
29 restraints	Δρ_min_ = −0.35 e Å^−^^3^

### Special details

**Table:**

Geometry. All e.s.d.'s (except the e.s.d. in the dihedral angle between two l.s. planes) are estimated using the full covariance matrix. The cell e.s.d.'s are taken into account individually in the estimation of e.s.d.'s in distances, angles and torsion angles; correlations between e.s.d.'s in cell parameters are only used when they are defined by crystal symmetry. An approximate (isotropic) treatment of cell e.s.d.'s is used for estimating e.s.d.'s involving l.s. planes.
Refinement. Refinement of *F*^2^ against ALL reflections. The weighted *R*-factor *wR* and goodness of fit *S* are based on *F*^2^, conventional *R*-factors *R* are based on *F*, with *F* set to zero for negative *F*^2^. The threshold expression of *F*^2^ > σ(*F*^2^) is used only for calculating *R*-factors(gt) *etc*. and is not relevant to the choice of reflections for refinement. *R*-factors based on *F*^2^ are statistically about twice as large as those based on *F*, and *R*- factors based on ALL data will be even larger.

### Fractional atomic coordinates and isotropic or equivalent isotropic displacement parameters (Å^2^)

**Table:**

	*x*	*y*	*z*	*U*_iso_*/*U*_eq_	
Li1	0.2089 (6)	0.3403 (9)	0.3594 (8)	0.038 (3)	
Li2	0.2083 (6)	0.3930 (7)	0.2230 (8)	0.036 (3)	
Li3	0.3031 (7)	0.4314 (8)	0.3137 (9)	0.042 (3)	
Li4	0.2925 (7)	0.2883 (8)	0.2619 (9)	0.043 (3)	
O1	0.1998 (2)	0.2886 (3)	0.2596 (3)	0.0329 (12)	
B1	0.1703 (4)	0.2205 (7)	0.2502 (6)	0.040 (2)	
O11	0.1002 (3)	0.0777 (4)	0.2420 (4)	0.0493 (16)	
C11	0.1805 (4)	0.1534 (4)	0.3043 (5)	0.036 (2)	
C12	0.1448 (4)	0.0847 (5)	0.2990 (5)	0.0366 (19)	
C13	0.1513 (5)	0.0228 (5)	0.3464 (6)	0.052 (3)	
H13	0.1263	−0.0212	0.3394	0.063*	
C14	0.1950 (5)	0.0258 (6)	0.4045 (6)	0.050 (2)	
H14	0.1996	−0.0163	0.4379	0.060*	
C15	0.2324 (5)	0.0898 (5)	0.4146 (6)	0.048 (2)	
H15	0.2629	0.0908	0.4536	0.058*	
C16	0.2245 (4)	0.1524 (5)	0.3669 (5)	0.040 (2)	
H16	0.2492	0.1964	0.3758	0.048*	
C21	0.1249 (4)	0.2014 (5)	0.1869 (5)	0.0375 (19)	
C22	0.0932 (4)	0.1308 (5)	0.1852 (5)	0.0359 (18)	
C23	0.0524 (5)	0.1086 (5)	0.1293 (6)	0.048 (2)	
H23	0.0321	0.0607	0.1314	0.057*	
C24	0.0420 (5)	0.1576 (6)	0.0708 (7)	0.060 (3)	
H24	0.0144	0.1423	0.0315	0.072*	
C25	0.0704 (5)	0.2309 (6)	0.0654 (6)	0.050 (2)	
H25	0.0619	0.2650	0.0247	0.060*	
C26	0.1102 (4)	0.2481 (5)	0.1228 (6)	0.046 (2)	
H26	0.1305	0.2960	0.1201	0.055*	
O2	0.2096 (2)	0.4444 (3)	0.3248 (3)	0.0355 (13)	
B2	0.1827 (4)	0.5118 (6)	0.3302 (6)	0.039 (2)	
O21	0.1125 (3)	0.6553 (4)	0.3318 (4)	0.0488 (16)	
C31	0.1916 (4)	0.5811 (5)	0.2722 (5)	0.042 (2)	
C32	0.1571 (3)	0.6410 (5)	0.2780 (6)	0.0376 (19)	
C33	0.1616 (6)	0.7047 (6)	0.2240 (8)	0.066 (3)	
H33	0.1344	0.7471	0.2271	0.079*	
C34	0.2070 (5)	0.7002 (6)	0.1690 (6)	0.051 (2)	
H34	0.2127	0.7411	0.1343	0.061*	
C35	0.2441 (5)	0.6365 (6)	0.1640 (6)	0.055 (3)	
H35	0.2757	0.6335	0.1263	0.066*	
C36	0.2351 (4)	0.5776 (5)	0.2139 (6)	0.046 (2)	
H36	0.2595	0.5329	0.2084	0.055*	
C41	0.1360 (4)	0.5356 (5)	0.3977 (5)	0.044 (2)	
C42	0.1061 (4)	0.6060 (5)	0.3915 (6)	0.048 (2)	
C43	0.0656 (4)	0.6295 (6)	0.4499 (7)	0.057 (3)	
H43	0.0449	0.6769	0.4453	0.069*	
C44	0.0554 (5)	0.5873 (6)	0.5113 (6)	0.050 (2)	
H44	0.0271	0.6043	0.5493	0.060*	
C45	0.0860 (5)	0.5181 (7)	0.5201 (6)	0.059 (3)	
H45	0.0798	0.4879	0.5643	0.071*	
C46	0.1261 (5)	0.4941 (6)	0.4624 (6)	0.051 (2)	
H46	0.1473	0.4471	0.4683	0.061*	
O3	0.3006 (2)	0.3929 (3)	0.2104 (3)	0.0352 (13)	
B3	0.3282 (5)	0.4009 (6)	0.1437 (5)	0.038 (2)	
O31	0.3956 (3)	0.4077 (4)	−0.0034 (4)	0.0457 (15)	
C51	0.3768 (4)	0.4645 (5)	0.1194 (5)	0.042 (2)	
C52	0.4062 (4)	0.4633 (5)	0.0504 (5)	0.037 (2)	
C53	0.4467 (4)	0.5175 (7)	0.0263 (6)	0.053 (3)	
H53	0.4633	0.5152	−0.0237	0.064*	
C54	0.4626 (5)	0.5721 (9)	0.0712 (9)	0.080 (4)	
H54	0.4922	0.6081	0.0538	0.095*	
C55	0.4373 (5)	0.5808 (7)	0.1468 (6)	0.060 (3)	
H55	0.4498	0.6206	0.1799	0.072*	
C56	0.3923 (5)	0.5255 (5)	0.1678 (7)	0.053 (3)	
H56	0.3721	0.5297	0.2158	0.063*	
C61	0.3153 (4)	0.3469 (5)	0.0745 (5)	0.0374 (19)	
C62	0.3486 (4)	0.3535 (5)	0.0062 (6)	0.042 (2)	
C63	0.3400 (5)	0.3044 (6)	−0.0551 (6)	0.051 (3)	
H63	0.3651	0.3095	−0.0993	0.062*	
C64	0.2947 (5)	0.2480 (6)	−0.0518 (7)	0.057 (3)	
H64	0.2883	0.2147	−0.0936	0.068*	
C65	0.2587 (5)	0.2414 (6)	0.0141 (6)	0.050 (2)	
H65	0.2271	0.2037	0.0166	0.060*	
C66	0.2681 (4)	0.2882 (5)	0.0753 (6)	0.042 (2)	
H66	0.2431	0.2819	0.1194	0.051*	
O4	0.3030 (3)	0.3267 (3)	0.3654 (3)	0.0372 (13)	
B4	0.3319 (4)	0.3156 (6)	0.4320 (5)	0.036 (2)	
O41	0.4020 (3)	0.3012 (5)	0.5751 (5)	0.065 (2)	
C71	0.3730 (4)	0.2444 (5)	0.4515 (6)	0.043 (2)	
C72	0.4030 (4)	0.2436 (6)	0.5220 (7)	0.055 (3)	
C73	0.4406 (5)	0.1772 (9)	0.5443 (9)	0.088 (6)	
H73	0.4606	0.1749	0.5924	0.105*	
C74	0.4458 (5)	0.1179 (7)	0.4918 (8)	0.066 (3)	
H74	0.4714	0.0756	0.5042	0.079*	
C75	0.4171 (5)	0.1182 (7)	0.4270 (7)	0.062 (3)	
H75	0.4205	0.0753	0.3944	0.075*	
C76	0.3810 (5)	0.1803 (6)	0.4040 (7)	0.054 (3)	
H76	0.3615	0.1794	0.3555	0.064*	
C81	0.3238 (4)	0.3718 (7)	0.5004 (6)	0.051 (3)	
C82	0.3607 (5)	0.3611 (6)	0.5690 (6)	0.050 (3)	
C83	0.3602 (7)	0.4080 (9)	0.6264 (7)	0.082 (4)	
H83	0.3870	0.4003	0.6687	0.099*	
C84	0.3187 (6)	0.4710 (9)	0.6244 (7)	0.077 (4)	
H84	0.3181	0.5048	0.6665	0.092*	
C85	0.2794 (6)	0.4849 (6)	0.5639 (6)	0.063 (3)	
H85	0.2507	0.5260	0.5634	0.075*	
C86	0.2850 (5)	0.4335 (7)	0.5031 (6)	0.056 (3)	
H86	0.2595	0.4424	0.4598	0.067*	
O100	0.1537 (3)	0.3001 (4)	0.4400 (4)	0.0420 (15)	
C101	0.0881 (5)	0.3054 (6)	0.4229 (7)	0.064 (3)	
H10A	0.0828	0.3345	0.3751	0.077*	
H10B	0.0672	0.3342	0.4640	0.077*	
C102	0.0568 (6)	0.2299 (7)	0.4144 (9)	0.088 (4)	
H10C	0.0127	0.2376	0.4022	0.131*	
H10D	0.0604	0.2015	0.4622	0.131*	
H10E	0.0769	0.2013	0.3734	0.131*	
C103	0.1706 (5)	0.2631 (6)	0.5096 (6)	0.054 (2)	
H10F	0.2164	0.2570	0.5108	0.065*	
H10G	0.1521	0.2116	0.5099	0.065*	
C104	0.1504 (7)	0.3042 (9)	0.5803 (7)	0.087 (4)	
H10H	0.1632	0.2751	0.6252	0.130*	
H10I	0.1051	0.3098	0.5802	0.130*	
H10J	0.1699	0.3545	0.5817	0.130*	
O200	0.1504 (3)	0.4389 (4)	0.1424 (4)	0.0443 (15)	
C201	0.1758 (5)	0.4729 (5)	0.0736 (5)	0.053 (2)	
H20A	0.2218	0.4709	0.0751	0.063*	
H20B	0.1630	0.5269	0.0705	0.063*	
C202	0.1523 (6)	0.4307 (7)	0.0048 (6)	0.069 (3)	
H20C	0.1692	0.4541	−0.0414	0.104*	
H20D	0.1068	0.4329	0.0035	0.104*	
H20E	0.1657	0.3775	0.0076	0.104*	
C203	0.0891 (5)	0.4639 (8)	0.1573 (6)	0.076 (4)	
H20F	0.0626	0.4556	0.1120	0.091*	
H20G	0.0892	0.5189	0.1692	0.091*	
C204	0.0638 (6)	0.4194 (10)	0.2243 (7)	0.100 (6)	
H20H	0.0214	0.4365	0.2357	0.150*	
H20I	0.0904	0.4277	0.2688	0.150*	
H20J	0.0632	0.3652	0.2117	0.150*	
O300	0.3595 (3)	0.5095 (4)	0.3595 (4)	0.0498 (16)	
C301	0.4210 (5)	0.4977 (8)	0.3876 (11)	0.095 (5)	
H30A	0.4468	0.5431	0.3775	0.114*	
H30B	0.4198	0.4891	0.4432	0.114*	
C302	0.4487 (5)	0.4300 (7)	0.3488 (7)	0.073 (4)	
H30C	0.4914	0.4223	0.3670	0.110*	
H30D	0.4492	0.4385	0.2937	0.110*	
H30E	0.4237	0.3849	0.3603	0.110*	
C303	0.3346 (5)	0.5812 (6)	0.3922 (6)	0.059 (3)	
H30F	0.2886	0.5792	0.3897	0.071*	
H30G	0.3463	0.5832	0.4467	0.071*	
C304	0.3556 (8)	0.6536 (6)	0.3554 (9)	0.099 (5)	
H30H	0.3366	0.6969	0.3816	0.149*	
H30I	0.3429	0.6538	0.3018	0.149*	
H30J	0.4011	0.6574	0.3586	0.149*	
O400	0.3445 (3)	0.2028 (4)	0.2106 (4)	0.0425 (14)	
C401	0.4073 (5)	0.2117 (6)	0.1855 (6)	0.059 (3)	
H40A	0.4074	0.2248	0.1307	0.071*	
H40B	0.4296	0.1629	0.1916	0.071*	
C402	0.4417 (5)	0.2725 (7)	0.2287 (8)	0.076 (4)	
H40C	0.4846	0.2756	0.2102	0.114*	
H40D	0.4418	0.2599	0.2831	0.114*	
H40E	0.4209	0.3215	0.2212	0.114*	
C403	0.3155 (5)	0.1381 (5)	0.1775 (7)	0.056 (3)	
H40F	0.3273	0.1350	0.1232	0.067*	
H40G	0.2697	0.1443	0.1803	0.067*	
C404	0.3335 (6)	0.0659 (5)	0.2164 (7)	0.067 (3)	
H40H	0.3111	0.0233	0.1932	0.101*	
H40I	0.3228	0.0692	0.2706	0.101*	
H40J	0.3784	0.0579	0.2111	0.101*	

### Atomic displacement parameters (Å^2^)

**Table:**

	*U*^11^	*U*^22^	*U*^33^	*U*^12^	*U*^13^	*U*^23^
Li1	0.031 (7)	0.051 (8)	0.030 (7)	0.002 (6)	0.004 (6)	−0.017 (6)
Li2	0.039 (7)	0.026 (6)	0.043 (8)	0.001 (6)	−0.020 (6)	−0.006 (6)
Li3	0.042 (7)	0.030 (7)	0.053 (9)	0.016 (6)	−0.018 (7)	−0.004 (6)
Li4	0.050 (8)	0.031 (7)	0.047 (8)	−0.020 (6)	−0.023 (7)	−0.007 (6)
O1	0.028 (3)	0.035 (3)	0.036 (3)	−0.004 (2)	−0.001 (2)	0.001 (2)
B1	0.018 (4)	0.061 (7)	0.042 (6)	0.003 (4)	0.006 (4)	0.008 (5)
O11	0.048 (4)	0.037 (3)	0.063 (4)	−0.010 (3)	0.005 (3)	−0.008 (3)
C11	0.033 (4)	0.025 (4)	0.049 (5)	−0.004 (3)	0.015 (4)	−0.005 (3)
C12	0.033 (4)	0.029 (4)	0.048 (5)	0.000 (4)	0.006 (4)	−0.009 (4)
C13	0.069 (7)	0.027 (5)	0.061 (7)	−0.017 (4)	0.015 (5)	−0.004 (4)
C14	0.049 (5)	0.043 (5)	0.057 (6)	−0.005 (4)	0.006 (5)	0.019 (5)
C15	0.050 (5)	0.046 (5)	0.048 (5)	0.003 (4)	0.012 (4)	0.010 (4)
C16	0.046 (5)	0.036 (4)	0.037 (5)	−0.004 (4)	0.002 (4)	0.006 (4)
C21	0.035 (4)	0.041 (4)	0.036 (5)	−0.010 (4)	0.004 (4)	−0.011 (4)
C22	0.025 (4)	0.036 (4)	0.047 (5)	−0.006 (3)	0.003 (4)	0.002 (4)
C23	0.050 (5)	0.043 (5)	0.049 (5)	−0.011 (4)	0.001 (5)	−0.007 (4)
C24	0.036 (5)	0.073 (7)	0.071 (7)	−0.016 (5)	−0.005 (5)	−0.012 (6)
C25	0.049 (6)	0.058 (6)	0.044 (6)	−0.007 (4)	−0.008 (4)	−0.018 (5)
C26	0.037 (5)	0.034 (5)	0.065 (6)	−0.003 (4)	−0.006 (4)	−0.004 (4)
O2	0.035 (3)	0.038 (3)	0.033 (3)	0.005 (2)	−0.010 (2)	−0.002 (2)
B2	0.024 (4)	0.047 (6)	0.046 (5)	−0.003 (4)	−0.003 (4)	−0.008 (5)
O21	0.046 (4)	0.043 (3)	0.058 (4)	0.002 (3)	−0.001 (3)	0.001 (3)
C31	0.038 (5)	0.049 (5)	0.038 (5)	−0.013 (4)	−0.013 (4)	0.004 (4)
C32	0.036 (4)	0.033 (5)	0.044 (6)	0.008 (4)	−0.007 (4)	0.006 (4)
C33	0.060 (7)	0.041 (6)	0.098 (10)	0.005 (5)	−0.025 (7)	0.001 (6)
C34	0.055 (6)	0.046 (5)	0.053 (6)	−0.009 (5)	0.006 (5)	0.002 (4)
C35	0.062 (7)	0.051 (5)	0.052 (6)	−0.002 (5)	0.011 (5)	0.002 (4)
C36	0.043 (5)	0.037 (5)	0.059 (6)	0.001 (4)	0.006 (5)	0.002 (4)
C41	0.044 (5)	0.048 (5)	0.040 (5)	−0.008 (4)	−0.003 (4)	−0.007 (4)
C42	0.037 (5)	0.041 (5)	0.065 (6)	−0.005 (4)	−0.004 (5)	−0.010 (4)
C43	0.039 (5)	0.064 (6)	0.068 (7)	0.005 (5)	0.012 (5)	−0.042 (6)
C44	0.049 (6)	0.052 (6)	0.049 (6)	0.001 (4)	0.005 (5)	−0.019 (5)
C45	0.052 (6)	0.075 (7)	0.050 (6)	−0.012 (5)	0.012 (5)	−0.024 (5)
C46	0.059 (6)	0.052 (5)	0.042 (5)	−0.003 (5)	0.009 (5)	−0.007 (4)
O3	0.032 (3)	0.043 (3)	0.031 (3)	−0.008 (2)	−0.003 (2)	−0.001 (2)
B3	0.031 (5)	0.055 (6)	0.029 (5)	0.016 (5)	−0.001 (4)	0.006 (4)
O31	0.033 (3)	0.056 (4)	0.048 (4)	−0.002 (3)	0.010 (3)	0.015 (3)
C51	0.036 (5)	0.046 (5)	0.045 (5)	0.004 (4)	−0.003 (4)	0.001 (4)
C52	0.025 (4)	0.048 (5)	0.037 (5)	0.001 (4)	−0.002 (3)	0.021 (4)
C53	0.030 (5)	0.080 (8)	0.049 (6)	−0.005 (5)	0.002 (4)	0.007 (5)
C54	0.043 (6)	0.098 (10)	0.098 (10)	0.000 (6)	−0.007 (7)	0.053 (8)
C55	0.049 (6)	0.074 (7)	0.058 (6)	−0.027 (5)	0.003 (5)	0.012 (5)
C56	0.049 (6)	0.038 (5)	0.071 (7)	−0.016 (4)	0.001 (5)	0.008 (5)
C61	0.041 (5)	0.040 (5)	0.032 (4)	0.007 (4)	−0.002 (4)	0.000 (4)
C62	0.038 (5)	0.042 (5)	0.045 (5)	0.013 (4)	−0.003 (4)	0.008 (4)
C63	0.050 (6)	0.064 (6)	0.040 (5)	0.008 (5)	0.002 (5)	−0.017 (5)
C64	0.056 (6)	0.046 (6)	0.068 (7)	0.015 (5)	−0.004 (5)	−0.018 (5)
C65	0.056 (6)	0.053 (5)	0.040 (5)	−0.001 (4)	0.008 (5)	−0.009 (4)
C66	0.047 (5)	0.039 (4)	0.041 (5)	−0.005 (4)	−0.001 (4)	0.003 (4)
O4	0.034 (3)	0.042 (3)	0.035 (3)	−0.004 (3)	−0.004 (2)	0.004 (3)
B4	0.032 (4)	0.052 (6)	0.023 (4)	−0.004 (4)	−0.008 (4)	0.010 (4)
O41	0.055 (4)	0.087 (5)	0.053 (4)	−0.024 (4)	−0.020 (4)	0.030 (4)
C71	0.015 (4)	0.064 (6)	0.050 (5)	−0.004 (4)	−0.003 (4)	0.028 (5)
C72	0.033 (5)	0.066 (7)	0.065 (7)	−0.007 (4)	0.002 (5)	0.028 (6)
C73	0.027 (5)	0.124 (12)	0.112 (12)	−0.004 (6)	−0.007 (6)	0.091 (11)
C74	0.051 (6)	0.058 (7)	0.089 (9)	0.015 (5)	0.009 (6)	0.030 (7)
C75	0.046 (6)	0.070 (7)	0.072 (8)	0.011 (5)	0.014 (6)	0.035 (6)
C76	0.038 (5)	0.055 (6)	0.068 (7)	0.016 (5)	0.006 (5)	0.011 (5)
C81	0.032 (5)	0.075 (7)	0.046 (6)	−0.015 (5)	−0.003 (4)	0.013 (5)
C82	0.056 (6)	0.058 (6)	0.035 (5)	−0.031 (5)	−0.017 (5)	0.011 (5)
C83	0.086 (9)	0.112 (11)	0.050 (7)	−0.047 (9)	−0.024 (6)	0.006 (7)
C84	0.073 (8)	0.111 (11)	0.047 (6)	−0.041 (8)	−0.006 (6)	−0.014 (7)
C85	0.072 (7)	0.063 (7)	0.053 (6)	−0.026 (5)	0.003 (5)	−0.023 (5)
C86	0.048 (6)	0.076 (7)	0.044 (6)	−0.012 (5)	−0.001 (5)	−0.010 (5)
O100	0.030 (3)	0.051 (4)	0.045 (3)	−0.004 (3)	0.000 (3)	0.009 (3)
C101	0.047 (6)	0.084 (8)	0.062 (7)	−0.003 (6)	0.004 (5)	0.006 (6)
C102	0.066 (8)	0.114 (11)	0.084 (9)	−0.008 (8)	0.018 (7)	−0.022 (8)
C103	0.047 (5)	0.066 (6)	0.049 (6)	0.003 (5)	0.015 (5)	0.007 (5)
C104	0.083 (9)	0.123 (12)	0.055 (7)	0.000 (9)	0.004 (7)	−0.009 (8)
O200	0.035 (3)	0.053 (4)	0.045 (4)	−0.002 (3)	−0.006 (3)	0.009 (3)
C201	0.053 (6)	0.050 (6)	0.055 (6)	0.008 (4)	−0.006 (5)	0.015 (5)
C202	0.074 (8)	0.091 (9)	0.043 (6)	−0.007 (7)	−0.014 (6)	0.015 (6)
C203	0.056 (7)	0.117 (11)	0.054 (7)	0.013 (7)	−0.013 (6)	−0.002 (7)
C204	0.040 (6)	0.197 (18)	0.063 (8)	−0.032 (8)	0.013 (6)	−0.038 (10)
O300	0.043 (3)	0.057 (4)	0.050 (4)	−0.013 (3)	−0.002 (3)	−0.005 (3)
C301	0.035 (6)	0.097 (10)	0.153 (16)	−0.009 (6)	−0.010 (7)	−0.035 (10)
C302	0.067 (7)	0.096 (9)	0.056 (7)	−0.009 (7)	−0.023 (6)	−0.004 (6)
C303	0.062 (7)	0.063 (7)	0.052 (6)	−0.009 (5)	−0.001 (5)	−0.014 (5)
C304	0.145 (14)	0.059 (8)	0.094 (10)	0.008 (9)	0.051 (10)	−0.002 (7)
O400	0.038 (3)	0.046 (3)	0.043 (3)	0.006 (3)	0.002 (3)	−0.002 (3)
C401	0.057 (6)	0.074 (7)	0.047 (6)	0.011 (5)	0.014 (5)	−0.011 (5)
C402	0.052 (7)	0.080 (8)	0.095 (10)	−0.024 (6)	0.026 (7)	−0.012 (7)
C403	0.058 (6)	0.046 (5)	0.063 (6)	0.002 (5)	0.005 (5)	−0.007 (5)
C404	0.085 (8)	0.042 (6)	0.074 (8)	0.016 (6)	−0.015 (7)	−0.018 (5)

### Geometric parameters (Å, °)

**Table:**

Li1—O2	1.932 (17)	C62—C63	1.392 (14)
Li1—O1	1.986 (14)	C63—C64	1.392 (15)
Li1—O100	1.980 (16)	C63—H63	0.9500
Li1—O4	2.037 (14)	C64—C65	1.397 (16)
Li1—Li2	2.57 (2)	C64—H64	0.9500
Li1—Li4	2.65 (2)	C65—C66	1.369 (14)
Li1—Li3	2.70 (2)	C65—H65	0.9500
Li2—O1	1.955 (14)	C66—H66	0.9500
Li2—O3	1.995 (15)	O4—B4	1.339 (10)
Li2—O2	2.005 (15)	B4—C71	1.572 (13)
Li2—O200	2.050 (14)	B4—C81	1.566 (16)
Li2—Li4	2.67 (2)	O41—C72	1.378 (14)
Li2—Li3	2.673 (18)	O41—C82	1.383 (14)
Li3—O3	1.938 (16)	C71—C72	1.397 (15)
Li3—O300	2.001 (16)	C71—C76	1.413 (15)
Li3—O2	2.031 (15)	C72—C73	1.473 (15)
Li3—O4	2.055 (16)	C73—C74	1.40 (2)
Li3—Li4	2.689 (19)	C73—H73	0.9500
Li4—O4	1.953 (15)	C74—C75	1.296 (19)
Li4—O1	1.991 (16)	C74—H74	0.9500
Li4—O3	2.058 (16)	C75—C76	1.400 (14)
Li4—O400	2.079 (17)	C75—H75	0.9500
O1—B1	1.367 (13)	C76—H76	0.9500
B1—C21	1.518 (14)	C81—C86	1.370 (16)
B1—C11	1.532 (14)	C81—C82	1.455 (14)
O11—C22	1.376 (11)	C82—C83	1.304 (19)
O11—C12	1.392 (11)	C83—C84	1.42 (2)
C11—C12	1.435 (11)	C83—H83	0.9500
C11—C16	1.450 (13)	C84—C85	1.381 (18)
C12—C13	1.379 (14)	C84—H84	0.9500
C13—C14	1.389 (16)	C85—C86	1.405 (15)
C13—H13	0.9500	C85—H85	0.9500
C14—C15	1.394 (14)	C86—H86	0.9500
C14—H14	0.9500	O100—C103	1.431 (13)
C15—C16	1.394 (13)	O100—C101	1.444 (12)
C15—H15	0.9500	C101—C102	1.496 (9)
C16—H16	0.9500	C101—H10A	0.9900
C21—C22	1.416 (11)	C101—H10B	0.9900
C21—C26	1.430 (14)	C102—H10C	0.9800
C22—C23	1.375 (13)	C102—H10D	0.9800
C23—C24	1.360 (16)	C102—H10E	0.9800
C23—H23	0.9500	C103—C104	1.503 (8)
C24—C25	1.431 (14)	C103—H10F	0.9900
C24—H24	0.9500	C103—H10G	0.9900
C25—C26	1.358 (14)	C104—H10H	0.9800
C25—H25	0.9500	C104—H10I	0.9800
C26—H26	0.9500	C104—H10J	0.9800
O2—B2	1.322 (12)	O200—C203	1.412 (13)
B2—C31	1.602 (15)	O200—C201	1.456 (12)
B2—C41	1.608 (14)	C201—C202	1.506 (8)
O21—C32	1.370 (11)	C201—H20A	0.9900
O21—C42	1.367 (13)	C201—H20B	0.9900
C31—C32	1.293 (13)	C202—H20C	0.9800
C31—C36	1.388 (14)	C202—H20D	0.9800
C32—C33	1.472 (15)	C202—H20E	0.9800
C33—C34	1.375 (17)	C203—C204	1.515 (9)
C33—H33	0.9500	C203—H20F	0.9900
C34—C35	1.379 (15)	C203—H20G	0.9900
C34—H34	0.9500	C204—H20H	0.9800
C35—C36	1.373 (14)	C204—H20I	0.9800
C35—H35	0.9500	C204—H20J	0.9800
C36—H36	0.9500	O300—C301	1.425 (14)
C41—C46	1.368 (14)	O300—C303	1.487 (13)
C41—C42	1.401 (13)	C301—C302	1.496 (8)
C42—C43	1.409 (14)	C301—H30A	0.9900
C43—C44	1.328 (16)	C301—H30B	0.9900
C43—H43	0.9500	C302—H30C	0.9800
C44—C45	1.394 (15)	C302—H30D	0.9800
C44—H44	0.9500	C302—H30E	0.9800
C45—C46	1.395 (15)	C303—C304	1.499 (8)
C45—H45	0.9500	C303—H30F	0.9900
C46—H46	0.9500	C303—H30G	0.9900
O3—B3	1.322 (11)	C304—H30H	0.9800
B3—C61	1.569 (14)	C304—H30I	0.9800
B3—C51	1.589 (15)	C304—H30J	0.9800
O31—C52	1.381 (12)	O400—C403	1.422 (12)
O31—C62	1.400 (12)	O400—C401	1.429 (12)
C51—C52	1.367 (13)	C401—C402	1.505 (8)
C51—C56	1.409 (14)	C401—H40A	0.9900
C52—C53	1.360 (13)	C401—H40B	0.9900
C53—C54	1.29 (2)	C402—H40C	0.9800
C53—H53	0.9500	C402—H40D	0.9800
C54—C55	1.44 (2)	C402—H40E	0.9800
C54—H54	0.9500	C403—C404	1.495 (8)
C55—C56	1.422 (13)	C403—H40F	0.9900
C55—H55	0.9500	C403—H40G	0.9900
C56—H56	0.9500	C404—H40H	0.9800
C61—C62	1.402 (14)	C404—H40I	0.9800
C61—C66	1.447 (13)	C404—H40J	0.9800
			
O2—Li1—O1	99.0 (7)	C56—C51—B3	122.0 (8)
O2—Li1—O100	124.7 (7)	C51—C52—C53	124.1 (10)
O1—Li1—O100	114.2 (7)	C51—C52—O31	122.9 (8)
O2—Li1—O4	96.9 (7)	C53—C52—O31	112.9 (8)
O1—Li1—O4	95.1 (6)	C54—C53—C52	120.1 (11)
O100—Li1—O4	121.1 (8)	C54—C53—H53	119.9
O2—Li1—Li2	50.5 (5)	C52—C53—H53	119.9
O1—Li1—Li2	48.8 (5)	C53—C54—C55	122.9 (11)
O100—Li1—Li2	142.5 (8)	C53—C54—H54	118.5
O4—Li1—Li2	95.5 (7)	C55—C54—H54	118.5
O2—Li1—Li4	96.9 (7)	C56—C55—C54	115.0 (11)
O1—Li1—Li4	48.4 (4)	C56—C55—H55	122.5
O100—Li1—Li4	138.3 (8)	C54—C55—H55	122.5
O4—Li1—Li4	47.1 (4)	C51—C56—C55	121.7 (10)
Li2—Li1—Li4	61.6 (6)	C51—C56—H56	119.2
O2—Li1—Li3	48.5 (4)	C55—C56—H56	119.2
O1—Li1—Li3	94.7 (7)	C62—C61—C66	115.1 (8)
O100—Li1—Li3	150.9 (7)	C62—C61—B3	121.5 (8)
O4—Li1—Li3	48.9 (4)	C66—C61—B3	123.4 (8)
Li2—Li1—Li3	60.9 (6)	C63—C62—C61	122.9 (9)
Li4—Li1—Li3	60.3 (5)	C63—C62—O31	115.1 (9)
O1—Li2—O3	97.3 (6)	C61—C62—O31	121.9 (9)
O1—Li2—O2	97.6 (7)	C62—C63—C64	120.2 (10)
O3—Li2—O2	94.9 (6)	C62—C63—H63	119.9
O1—Li2—O200	122.8 (7)	C64—C63—H63	119.9
O3—Li2—O200	121.8 (8)	C63—C64—C65	118.7 (10)
O2—Li2—O200	116.5 (7)	C63—C64—H64	120.6
O1—Li2—Li1	49.8 (5)	C65—C64—H64	120.6
O3—Li2—Li1	95.6 (6)	C66—C65—C64	121.2 (10)
O2—Li2—Li1	48.0 (5)	C66—C65—H65	119.4
O200—Li2—Li1	142.2 (8)	C64—C65—H65	119.4
O1—Li2—Li4	48.0 (5)	C65—C66—C61	121.7 (9)
O3—Li2—Li4	49.8 (4)	C65—C66—H66	119.1
O2—Li2—Li4	94.2 (6)	C61—C66—H66	119.1
O200—Li2—Li4	149.2 (7)	B4—O4—Li4	144.8 (8)
Li1—Li2—Li4	60.6 (6)	B4—O4—Li1	121.4 (7)
O1—Li2—Li3	96.5 (6)	Li4—O4—Li1	83.0 (6)
O3—Li2—Li3	46.3 (4)	B4—O4—Li3	121.1 (7)
O2—Li2—Li3	48.9 (5)	Li4—O4—Li3	84.2 (6)
O200—Li2—Li3	140.7 (7)	Li1—O4—Li3	82.7 (7)
Li1—Li2—Li3	62.0 (6)	O4—B4—C71	124.5 (8)
Li4—Li2—Li3	60.4 (5)	O4—B4—C81	121.9 (9)
O3—Li3—O300	129.4 (9)	C71—B4—C81	113.5 (8)
O3—Li3—O2	95.8 (6)	C72—O41—C82	121.2 (8)
O300—Li3—O2	118.8 (7)	C72—C71—C76	117.3 (9)
O3—Li3—O4	95.7 (6)	C72—C71—B4	117.4 (9)
O300—Li3—O4	116.1 (7)	C76—C71—B4	125.2 (8)
O2—Li3—O4	93.3 (7)	O41—C72—C71	125.9 (9)
O3—Li3—Li2	48.1 (5)	O41—C72—C73	114.3 (11)
O300—Li3—Li2	151.0 (7)	C71—C72—C73	119.8 (12)
O2—Li3—Li2	48.1 (5)	C74—C73—C72	117.5 (12)
O4—Li3—Li2	92.0 (6)	C74—C73—H73	121.3
O3—Li3—Li4	49.6 (5)	C72—C73—H73	121.3
O300—Li3—Li4	146.3 (7)	C75—C74—C73	122.6 (11)
O2—Li3—Li4	93.1 (7)	C75—C74—H74	118.7
O4—Li3—Li4	46.3 (4)	C73—C74—H74	118.7
Li2—Li3—Li4	59.8 (5)	C74—C75—C76	121.3 (13)
O3—Li3—Li1	92.8 (6)	C74—C75—H75	119.3
O300—Li3—Li1	137.8 (8)	C76—C75—H75	119.3
O2—Li3—Li1	45.5 (5)	C75—C76—C71	121.4 (11)
O4—Li3—Li1	48.4 (5)	C75—C76—H76	119.3
Li2—Li3—Li1	57.1 (5)	C71—C76—H76	119.3
Li4—Li3—Li1	58.8 (6)	C86—C81—C82	113.9 (10)
O4—Li4—O1	97.6 (8)	C86—C81—B4	126.5 (9)
O4—Li4—O3	95.1 (6)	C82—C81—B4	119.6 (10)
O1—Li4—O3	94.1 (7)	C83—C82—O41	115.3 (11)
O4—Li4—O400	126.3 (8)	C83—C82—C81	123.7 (13)
O1—Li4—O400	122.0 (6)	O41—C82—C81	120.9 (10)
O3—Li4—O400	114.4 (8)	C82—C83—C84	118.7 (12)
O4—Li4—Li1	49.8 (5)	C82—C83—H83	120.7
O1—Li4—Li1	48.2 (5)	C84—C83—H83	120.7
O3—Li4—Li1	91.8 (7)	C85—C84—C83	122.7 (12)
O400—Li4—Li1	153.6 (8)	C85—C84—H84	118.6
O4—Li4—Li2	94.4 (7)	C83—C84—H84	118.6
O1—Li4—Li2	46.8 (5)	C84—C85—C86	114.8 (12)
O3—Li4—Li2	47.7 (5)	C84—C85—H85	122.6
O400—Li4—Li2	138.8 (7)	C86—C85—H85	122.6
Li1—Li4—Li2	57.8 (6)	C81—C86—C85	126.1 (11)
O4—Li4—Li3	49.5 (5)	C81—C86—H86	117.0
O1—Li4—Li3	95.1 (7)	C85—C86—H86	117.0
O3—Li4—Li3	45.8 (5)	C103—O100—C101	117.1 (7)
O400—Li4—Li3	141.2 (8)	C103—O100—Li1	128.4 (7)
Li1—Li4—Li3	60.9 (6)	C101—O100—Li1	114.3 (7)
Li2—Li4—Li3	59.8 (5)	O100—C101—C102	113.6 (10)
B1—O1—Li2	145.9 (7)	O100—C101—H10A	108.8
B1—O1—Li1	123.7 (7)	C102—C101—H10A	108.8
Li2—O1—Li1	81.4 (6)	O100—C101—H10B	108.8
B1—O1—Li4	117.6 (7)	C102—C101—H10B	108.8
Li2—O1—Li4	85.2 (6)	H10A—C101—H10B	107.7
Li1—O1—Li4	83.4 (6)	C101—C102—H10C	109.5
O1—B1—C21	125.5 (9)	C101—C102—H10D	109.5
O1—B1—C11	122.4 (9)	H10C—C102—H10D	109.5
C21—B1—C11	112.1 (9)	C101—C102—H10E	109.5
C22—O11—C12	122.4 (7)	H10C—C102—H10E	109.5
C12—C11—C16	112.8 (8)	H10D—C102—H10E	109.5
C12—C11—B1	122.2 (8)	O100—C103—C104	114.4 (10)
C16—C11—B1	125.0 (8)	O100—C103—H10F	108.7
C13—C12—O11	115.7 (8)	C104—C103—H10F	108.7
C13—C12—C11	125.0 (9)	O100—C103—H10G	108.7
O11—C12—C11	119.4 (8)	C104—C103—H10G	108.7
C14—C13—C12	118.8 (9)	H10F—C103—H10G	107.6
C14—C13—H13	120.6	C103—C104—H10H	109.5
C12—C13—H13	120.6	C103—C104—H10I	109.5
C13—C14—C15	120.9 (9)	H10H—C104—H10I	109.5
C13—C14—H14	119.5	C103—C104—H10J	109.5
C15—C14—H14	119.5	H10H—C104—H10J	109.5
C14—C15—C16	119.5 (10)	H10I—C104—H10J	109.5
C14—C15—H15	120.2	C203—O200—C201	112.0 (7)
C16—C15—H15	120.2	C203—O200—Li2	124.1 (8)
C15—C16—C11	122.9 (9)	C201—O200—Li2	120.6 (7)
C15—C16—H16	118.5	O200—C201—C202	109.8 (8)
C11—C16—H16	118.5	O200—C201—H20A	109.7
C22—C21—C26	112.5 (8)	C202—C201—H20A	109.7
C22—C21—B1	121.2 (9)	O200—C201—H20B	109.7
C26—C21—B1	126.2 (8)	C202—C201—H20B	109.7
O11—C22—C23	113.2 (7)	H20A—C201—H20B	108.2
O11—C22—C21	122.0 (8)	C201—C202—H20C	109.5
C23—C22—C21	124.8 (9)	C201—C202—H20D	109.5
C24—C23—C22	117.6 (9)	H20C—C202—H20D	109.5
C24—C23—H23	121.2	C201—C202—H20E	109.5
C22—C23—H23	121.2	H20C—C202—H20E	109.5
C23—C24—C25	123.6 (10)	H20D—C202—H20E	109.5
C23—C24—H24	118.2	O200—C203—C204	108.5 (9)
C25—C24—H24	118.2	O200—C203—H20F	110.0
C26—C25—C24	114.8 (10)	C204—C203—H20F	110.0
C26—C25—H25	122.6	O200—C203—H20G	110.0
C24—C25—H25	122.6	C204—C203—H20G	110.0
C25—C26—C21	126.6 (9)	H20F—C203—H20G	108.4
C25—C26—H26	116.7	C203—C204—H20H	109.5
C21—C26—H26	116.7	C203—C204—H20I	109.5
B2—O2—Li1	145.6 (7)	H20H—C204—H20I	109.5
B2—O2—Li2	117.6 (7)	C203—C204—H20J	109.5
Li1—O2—Li2	81.5 (6)	H20H—C204—H20J	109.5
B2—O2—Li3	122.7 (7)	H20I—C204—H20J	109.5
Li1—O2—Li3	86.0 (6)	C301—O300—C303	108.9 (8)
Li2—O2—Li3	83.0 (6)	C301—O300—Li3	126.8 (8)
O2—B2—C31	125.8 (9)	C303—O300—Li3	121.4 (7)
O2—B2—C41	124.0 (9)	C302—C301—O300	109.1 (10)
C31—B2—C41	110.2 (8)	C302—C301—H30A	109.9
C32—O21—C42	118.8 (7)	O300—C301—H30A	109.9
C32—C31—C36	118.8 (9)	C302—C301—H30B	109.9
C32—C31—B2	120.2 (9)	O300—C301—H30B	109.9
C36—C31—B2	121.0 (9)	H30A—C301—H30B	108.3
C31—C32—O21	127.3 (9)	C301—C302—H30C	109.5
C31—C32—C33	122.2 (9)	C301—C302—H30D	109.5
O21—C32—C33	110.6 (8)	H30C—C302—H30D	109.5
C34—C33—C32	117.2 (10)	C301—C302—H30E	109.5
C34—C33—H33	121.4	H30C—C302—H30E	109.5
C32—C33—H33	121.4	H30D—C302—H30E	109.5
C35—C34—C33	120.1 (10)	C304—C303—O300	116.5 (9)
C35—C34—H34	120.0	C304—C303—H30F	108.2
C33—C34—H34	120.0	O300—C303—H30F	108.2
C36—C35—C34	119.5 (10)	C304—C303—H30G	108.2
C36—C35—H35	120.2	O300—C303—H30G	108.2
C34—C35—H35	120.2	H30F—C303—H30G	107.3
C35—C36—C31	122.1 (9)	C303—C304—H30H	109.5
C35—C36—H36	118.9	C303—C304—H30I	109.5
C31—C36—H36	118.9	H30H—C304—H30I	109.5
C46—C41—C42	117.7 (9)	C303—C304—H30J	109.5
C46—C41—B2	124.8 (9)	H30H—C304—H30J	109.5
C42—C41—B2	117.4 (9)	H30I—C304—H30J	109.5
O21—C42—C41	125.1 (9)	C403—O400—C401	112.1 (7)
O21—C42—C43	115.7 (9)	C403—O400—Li4	121.4 (7)
C41—C42—C43	119.1 (10)	C401—O400—Li4	124.1 (7)
C44—C43—C42	122.0 (10)	O400—C401—C402	112.7 (8)
C44—C43—H43	119.0	O400—C401—H40A	109.1
C42—C43—H43	119.0	C402—C401—H40A	109.1
C43—C44—C45	120.1 (10)	O400—C401—H40B	109.1
C43—C44—H44	120.0	C402—C401—H40B	109.1
C45—C44—H44	120.0	H40A—C401—H40B	107.8
C44—C45—C46	118.4 (11)	C401—C402—H40C	109.5
C44—C45—H45	120.8	C401—C402—H40D	109.5
C46—C45—H45	120.8	H40C—C402—H40D	109.5
C41—C46—C45	122.6 (10)	C401—C402—H40E	109.5
C41—C46—H46	118.7	H40C—C402—H40E	109.5
C45—C46—H46	118.7	H40D—C402—H40E	109.5
B3—O3—Li3	141.3 (8)	O400—C403—C404	112.4 (9)
B3—O3—Li2	123.0 (7)	O400—C403—H40F	109.1
Li3—O3—Li2	85.6 (6)	C404—C403—H40F	109.1
B3—O3—Li4	121.5 (7)	O400—C403—H40G	109.1
Li3—O3—Li4	84.5 (6)	C404—C403—H40G	109.1
Li2—O3—Li4	82.5 (6)	H40F—C403—H40G	107.9
O3—B3—C61	122.9 (9)	C403—C404—H40H	109.5
O3—B3—C51	127.5 (9)	C403—C404—H40I	109.5
C61—B3—C51	109.6 (8)	H40H—C404—H40I	109.5
C52—O31—C62	121.3 (7)	C403—C404—H40J	109.5
C52—C51—C56	116.0 (9)	H40H—C404—H40J	109.5
C52—C51—B3	122.0 (8)	H40I—C404—H40J	109.5
			
O2—Li1—Li2—O1	−173.6 (7)	O200—Li2—O2—Li1	137.6 (8)
O100—Li1—Li2—O1	−75.7 (12)	Li4—Li2—O2—Li1	−43.2 (5)
O4—Li1—Li2—O1	92.0 (6)	Li3—Li2—O2—Li1	−87.0 (6)
Li4—Li1—Li2—O1	58.0 (5)	O1—Li2—O2—Li3	91.9 (6)
Li3—Li1—Li2—O1	127.9 (6)	O3—Li2—O2—Li3	−6.1 (6)
O2—Li1—Li2—O3	91.5 (6)	O200—Li2—O2—Li3	−135.4 (8)
O1—Li1—Li2—O3	−94.9 (6)	Li1—Li2—O2—Li3	87.0 (6)
O100—Li1—Li2—O3	−170.5 (11)	Li4—Li2—O2—Li3	43.8 (6)
O4—Li1—Li2—O3	−2.9 (6)	O3—Li3—O2—B2	−112.1 (8)
Li4—Li1—Li2—O3	−36.9 (5)	O300—Li3—O2—B2	29.6 (13)
Li3—Li1—Li2—O3	33.1 (5)	O4—Li3—O2—B2	151.8 (7)
O1—Li1—Li2—O2	173.6 (7)	Li2—Li3—O2—B2	−118.4 (9)
O100—Li1—Li2—O2	97.9 (12)	Li4—Li3—O2—B2	−161.8 (8)
O4—Li1—Li2—O2	−94.4 (6)	Li1—Li3—O2—B2	159.7 (9)
Li4—Li1—Li2—O2	−128.4 (6)	O3—Li3—O2—Li1	88.2 (7)
Li3—Li1—Li2—O2	−58.5 (5)	O300—Li3—O2—Li1	−130.1 (9)
O2—Li1—Li2—O200	−79.7 (11)	O4—Li3—O2—Li1	−7.9 (6)
O1—Li1—Li2—O200	93.9 (11)	Li2—Li3—O2—Li1	81.9 (6)
O100—Li1—Li2—O200	18.2 (19)	Li4—Li3—O2—Li1	38.5 (6)
O4—Li1—Li2—O200	−174.1 (9)	O3—Li3—O2—Li2	6.3 (6)
Li4—Li1—Li2—O200	151.9 (11)	O300—Li3—O2—Li2	147.9 (9)
Li3—Li1—Li2—O200	−138.2 (12)	O4—Li3—O2—Li2	−89.8 (6)
O2—Li1—Li2—Li4	128.4 (6)	Li4—Li3—O2—Li2	−43.4 (6)
O1—Li1—Li2—Li4	−58.0 (5)	Li1—Li3—O2—Li2	−81.9 (6)
O100—Li1—Li2—Li4	−133.7 (12)	Li1—O2—B2—C31	−164.6 (9)
O4—Li1—Li2—Li4	34.0 (5)	Li2—O2—B2—C31	−46.7 (11)
Li3—Li1—Li2—Li4	69.9 (5)	Li3—O2—B2—C31	53.2 (12)
O2—Li1—Li2—Li3	58.5 (5)	Li1—O2—B2—C41	15.8 (17)
O1—Li1—Li2—Li3	−127.9 (6)	Li2—O2—B2—C41	133.6 (9)
O100—Li1—Li2—Li3	156.4 (12)	Li3—O2—B2—C41	−126.5 (9)
O4—Li1—Li2—Li3	−35.9 (5)	O2—B2—C31—C32	171.5 (9)
Li4—Li1—Li2—Li3	−69.9 (5)	C41—B2—C31—C32	−8.8 (11)
O1—Li2—Li3—O3	94.0 (7)	O2—B2—C31—C36	−7.0 (14)
O2—Li2—Li3—O3	−171.5 (8)	C41—B2—C31—C36	172.7 (8)
O200—Li2—Li3—O3	−88.9 (13)	C36—C31—C32—O21	−179.2 (9)
Li1—Li2—Li3—O3	131.3 (7)	B2—C31—C32—O21	2.3 (14)
Li4—Li2—Li3—O3	61.0 (6)	C36—C31—C32—C33	1.2 (14)
O1—Li2—Li3—O300	−168.0 (16)	B2—C31—C32—C33	−177.2 (8)
O3—Li2—Li3—O300	98.0 (19)	C42—O21—C32—C31	7.2 (14)
O2—Li2—Li3—O300	−73.5 (17)	C42—O21—C32—C33	−173.2 (8)
O200—Li2—Li3—O300	9(3)	C31—C32—C33—C34	−3.7 (15)
Li1—Li2—Li3—O300	−130.7 (18)	O21—C32—C33—C34	176.7 (9)
Li4—Li2—Li3—O300	159.0 (19)	C32—C33—C34—C35	2.7 (15)
O1—Li2—Li3—O2	−94.5 (7)	C33—C34—C35—C36	0.5 (16)
O3—Li2—Li3—O2	171.5 (8)	C34—C35—C36—C31	−3.2 (16)
O200—Li2—Li3—O2	82.6 (13)	C32—C31—C36—C35	2.3 (15)
Li1—Li2—Li3—O2	−57.2 (5)	B2—C31—C36—C35	−179.3 (9)
Li4—Li2—Li3—O2	−127.4 (8)	O2—B2—C41—C46	10.3 (14)
O1—Li2—Li3—O4	−1.9 (7)	C31—B2—C41—C46	−169.4 (9)
O3—Li2—Li3—O4	−95.9 (6)	O2—B2—C41—C42	−173.5 (9)
O2—Li2—Li3—O4	92.6 (7)	C31—B2—C41—C42	6.8 (11)
O200—Li2—Li3—O4	175.2 (11)	C32—O21—C42—C41	−9.1 (13)
Li1—Li2—Li3—O4	35.4 (6)	C32—O21—C42—C43	171.5 (8)
Li4—Li2—Li3—O4	−34.8 (5)	C46—C41—C42—O21	178.0 (9)
O1—Li2—Li3—Li4	32.9 (6)	B2—C41—C42—O21	1.6 (13)
O3—Li2—Li3—Li4	−61.0 (6)	C46—C41—C42—C43	−2.6 (14)
O2—Li2—Li3—Li4	127.4 (8)	B2—C41—C42—C43	−179.1 (8)
O200—Li2—Li3—Li4	−150.0 (13)	O21—C42—C43—C44	−179.7 (9)
Li1—Li2—Li3—Li4	70.2 (6)	C41—C42—C43—C44	0.9 (14)
O1—Li2—Li3—Li1	−37.3 (5)	C42—C43—C44—C45	1.1 (16)
O3—Li2—Li3—Li1	−131.3 (7)	C43—C44—C45—C46	−1.4 (16)
O2—Li2—Li3—Li1	57.2 (5)	C42—C41—C46—C45	2.4 (15)
O200—Li2—Li3—Li1	139.8 (14)	B2—C41—C46—C45	178.6 (9)
Li4—Li2—Li3—Li1	−70.2 (6)	C44—C45—C46—C41	−0.4 (16)
O2—Li1—Li3—O3	−95.4 (7)	O300—Li3—O3—B3	−0.5 (17)
O1—Li1—Li3—O3	2.5 (7)	O2—Li3—O3—B3	134.7 (10)
O100—Li1—Li3—O3	176.0 (15)	O4—Li3—O3—B3	−131.3 (10)
O4—Li1—Li3—O3	95.1 (6)	Li2—Li3—O3—B3	141.1 (12)
Li2—Li1—Li3—O3	−34.0 (5)	Li4—Li3—O3—B3	−136.1 (12)
Li4—Li1—Li3—O3	38.0 (5)	Li1—Li3—O3—B3	−179.8 (10)
O2—Li1—Li3—O300	85.5 (10)	O300—Li3—O3—Li2	−141.6 (9)
O1—Li1—Li3—O300	−176.6 (9)	O2—Li3—O3—Li2	−6.3 (6)
O100—Li1—Li3—O300	−3(2)	O4—Li3—O3—Li2	87.6 (7)
O4—Li1—Li3—O300	−84.0 (10)	Li4—Li3—O3—Li2	82.8 (6)
Li2—Li1—Li3—O300	146.8 (10)	Li1—Li3—O3—Li2	39.2 (6)
Li4—Li1—Li3—O300	−141.1 (11)	O300—Li3—O3—Li4	135.6 (10)
O1—Li1—Li3—O2	97.9 (7)	O2—Li3—O3—Li4	−89.2 (7)
O100—Li1—Li3—O2	−88.6 (16)	O4—Li3—O3—Li4	4.8 (6)
O4—Li1—Li3—O2	−169.5 (8)	Li2—Li3—O3—Li4	−82.8 (6)
Li2—Li1—Li3—O2	61.3 (5)	Li1—Li3—O3—Li4	−43.7 (6)
Li4—Li1—Li3—O2	133.4 (7)	O1—Li2—O3—B3	116.0 (8)
O2—Li1—Li3—O4	169.5 (8)	O2—Li2—O3—B3	−145.6 (8)
O1—Li1—Li3—O4	−92.7 (6)	O200—Li2—O3—B3	−20.2 (12)
O100—Li1—Li3—O4	80.9 (16)	Li1—Li2—O3—B3	166.1 (8)
Li2—Li1—Li3—O4	−129.2 (7)	Li4—Li2—O3—B3	122.9 (9)
Li4—Li1—Li3—O4	−57.2 (5)	Li3—Li2—O3—B3	−152.1 (10)
O2—Li1—Li3—Li2	−61.3 (5)	O1—Li2—O3—Li3	−92.0 (7)
O1—Li1—Li3—Li2	36.5 (5)	O2—Li2—O3—Li3	6.4 (6)
O100—Li1—Li3—Li2	−149.9 (17)	O200—Li2—O3—Li3	131.9 (8)
O4—Li1—Li3—Li2	129.2 (7)	Li1—Li2—O3—Li3	−41.8 (6)
Li4—Li1—Li3—Li2	72.0 (6)	Li4—Li2—O3—Li3	−85.0 (6)
O2—Li1—Li3—Li4	−133.4 (7)	O1—Li2—O3—Li4	−6.9 (6)
O1—Li1—Li3—Li4	−35.5 (6)	O2—Li2—O3—Li4	91.4 (7)
O100—Li1—Li3—Li4	138.1 (17)	O200—Li2—O3—Li4	−143.1 (8)
O4—Li1—Li3—Li4	57.2 (5)	Li1—Li2—O3—Li4	43.2 (6)
Li2—Li1—Li3—Li4	−72.0 (6)	Li3—Li2—O3—Li4	85.0 (6)
O2—Li1—Li4—O4	93.1 (7)	O4—Li4—O3—B3	144.4 (7)
O1—Li1—Li4—O4	−170.9 (9)	O1—Li4—O3—B3	−117.5 (8)
O100—Li1—Li4—O4	−90.9 (11)	O400—Li4—O3—B3	10.5 (10)
Li2—Li1—Li4—O4	130.6 (7)	Li1—Li4—O3—B3	−165.8 (7)
Li3—Li1—Li4—O4	59.8 (5)	Li2—Li4—O3—B3	−124.3 (8)
O2—Li1—Li4—O1	−96.0 (7)	Li3—Li4—O3—B3	149.4 (8)
O100—Li1—Li4—O1	80.0 (11)	O4—Li4—O3—Li3	−5.0 (7)
O4—Li1—Li4—O1	170.9 (9)	O1—Li4—O3—Li3	93.1 (7)
Li2—Li1—Li4—O1	−58.5 (5)	O400—Li4—O3—Li3	−138.9 (8)
Li3—Li1—Li4—O1	−129.3 (8)	Li1—Li4—O3—Li3	44.8 (6)
O2—Li1—Li4—O3	−2.1 (6)	Li2—Li4—O3—Li3	86.3 (6)
O1—Li1—Li4—O3	93.9 (7)	O4—Li4—O3—Li2	−91.3 (7)
O100—Li1—Li4—O3	173.9 (10)	O1—Li4—O3—Li2	6.8 (6)
O4—Li1—Li4—O3	−95.2 (6)	O400—Li4—O3—Li2	134.8 (7)
Li2—Li1—Li4—O3	35.4 (5)	Li1—Li4—O3—Li2	−41.5 (5)
Li3—Li1—Li4—O3	−35.4 (5)	Li3—Li4—O3—Li2	−86.3 (6)
O2—Li1—Li4—O400	−174.5 (15)	Li3—O3—B3—C61	171.5 (9)
O1—Li1—Li4—O400	−78.4 (16)	Li2—O3—B3—C61	−56.9 (12)
O100—Li1—Li4—O400	2(2)	Li4—O3—B3—C61	45.6 (11)
O4—Li1—Li4—O400	92.5 (17)	Li3—O3—B3—C51	−9.9 (16)
Li2—Li1—Li4—O400	−137.0 (16)	Li2—O3—B3—C51	121.7 (9)
Li3—Li1—Li4—O400	152.3 (17)	Li4—O3—B3—C51	−135.8 (9)
O2—Li1—Li4—Li2	−37.5 (5)	O3—B3—C51—C52	174.5 (8)
O1—Li1—Li4—Li2	58.5 (5)	C61—B3—C51—C52	−6.8 (11)
O100—Li1—Li4—Li2	138.5 (12)	O3—B3—C51—C56	−5.3 (14)
O4—Li1—Li4—Li2	−130.6 (7)	C61—B3—C51—C56	173.4 (8)
Li3—Li1—Li4—Li2	−70.8 (6)	C56—C51—C52—C53	−2.1 (13)
O2—Li1—Li4—Li3	33.3 (5)	B3—C51—C52—C53	178.1 (8)
O1—Li1—Li4—Li3	129.3 (8)	C56—C51—C52—O31	−179.4 (8)
O100—Li1—Li4—Li3	−150.7 (11)	B3—C51—C52—O31	0.7 (12)
O4—Li1—Li4—Li3	−59.8 (5)	C62—O31—C52—C51	7.2 (11)
Li2—Li1—Li4—Li3	70.8 (6)	C62—O31—C52—C53	−170.4 (8)
O1—Li2—Li4—O4	−96.3 (8)	C51—C52—C53—C54	4.4 (15)
O3—Li2—Li4—O4	92.9 (6)	O31—C52—C53—C54	−178.0 (10)
O2—Li2—Li4—O4	0.1 (7)	C52—C53—C54—C55	−2.5 (17)
O200—Li2—Li4—O4	178.8 (13)	C53—C54—C55—C56	−1.2 (17)
Li1—Li2—Li4—O4	−35.6 (6)	C52—C51—C56—C55	−1.9 (14)
Li3—Li2—Li4—O4	37.0 (6)	B3—C51—C56—C55	177.9 (9)
O3—Li2—Li4—O1	−170.7 (8)	C54—C55—C56—C51	3.5 (16)
O2—Li2—Li4—O1	96.5 (7)	O3—B3—C61—C62	−175.3 (8)
O200—Li2—Li4—O1	−84.9 (15)	C51—B3—C61—C62	5.9 (11)
Li1—Li2—Li4—O1	60.7 (5)	O3—B3—C61—C66	5.8 (13)
Li3—Li2—Li4—O1	133.4 (8)	C51—B3—C61—C66	−173.0 (8)
O1—Li2—Li4—O3	170.7 (8)	C66—C61—C62—C63	−3.7 (13)
O2—Li2—Li4—O3	−92.8 (6)	B3—C61—C62—C63	177.3 (9)
O200—Li2—Li4—O3	85.9 (14)	C66—C61—C62—O31	179.9 (7)
Li1—Li2—Li4—O3	−128.5 (7)	B3—C61—C62—O31	0.9 (13)
Li3—Li2—Li4—O3	−55.9 (5)	C52—O31—C62—C63	175.3 (8)
O1—Li2—Li4—O400	91.8 (10)	C52—O31—C62—C61	−8.0 (12)
O3—Li2—Li4—O400	−78.9 (11)	C61—C62—C63—C64	3.1 (15)
O2—Li2—Li4—O400	−171.7 (10)	O31—C62—C63—C64	179.8 (8)
O200—Li2—Li4—O400	7(2)	C62—C63—C64—C65	−0.5 (16)
Li1—Li2—Li4—O400	152.5 (11)	C63—C64—C65—C66	−1.2 (16)
Li3—Li2—Li4—O400	−134.8 (12)	C64—C65—C66—C61	0.4 (15)
O1—Li2—Li4—Li1	−60.7 (5)	C62—C61—C66—C65	1.9 (13)
O3—Li2—Li4—Li1	128.5 (7)	B3—C61—C66—C65	−179.1 (9)
O2—Li2—Li4—Li1	35.7 (5)	O1—Li4—O4—B4	130.7 (11)
O200—Li2—Li4—Li1	−145.6 (16)	O3—Li4—O4—B4	−134.4 (11)
Li3—Li2—Li4—Li1	72.6 (7)	O400—Li4—O4—B4	−9.0 (18)
O1—Li2—Li4—Li3	−133.4 (8)	Li1—Li4—O4—B4	137.5 (13)
O3—Li2—Li4—Li3	55.9 (5)	Li2—Li4—O4—B4	177.6 (11)
O2—Li2—Li4—Li3	−36.9 (6)	Li3—Li4—O4—B4	−139.2 (13)
O200—Li2—Li4—Li3	141.8 (15)	O1—Li4—O4—Li1	−6.8 (7)
Li1—Li2—Li4—Li3	−72.6 (7)	O3—Li4—O4—Li1	88.1 (7)
O3—Li3—Li4—O4	173.4 (9)	O400—Li4—O4—Li1	−146.5 (10)
O300—Li3—Li4—O4	70.3 (14)	Li2—Li4—O4—Li1	40.1 (6)
O2—Li3—Li4—O4	−91.6 (7)	Li3—Li4—O4—Li1	83.3 (7)
Li2—Li3—Li4—O4	−127.9 (8)	O1—Li4—O4—Li3	−90.2 (7)
Li1—Li3—Li4—O4	−60.3 (6)	O3—Li4—O4—Li3	4.7 (6)
O3—Li3—Li4—O1	−90.8 (6)	O400—Li4—O4—Li3	130.1 (10)
O300—Li3—Li4—O1	166.0 (14)	Li1—Li4—O4—Li3	−83.3 (7)
O2—Li3—Li4—O1	4.1 (7)	Li2—Li4—O4—Li3	−43.2 (6)
O4—Li3—Li4—O1	95.7 (7)	O2—Li1—O4—B4	114.2 (8)
Li2—Li3—Li4—O1	−32.1 (6)	O1—Li1—O4—B4	−146.0 (7)
Li1—Li3—Li4—O1	35.4 (6)	O100—Li1—O4—B4	−23.8 (12)
O300—Li3—Li4—O3	−103.1 (16)	Li2—Li1—O4—B4	165.0 (7)
O2—Li3—Li4—O3	95.0 (6)	Li4—Li1—O4—B4	−152.9 (9)
O4—Li3—Li4—O3	−173.4 (9)	Li3—Li1—O4—B4	122.1 (9)
Li2—Li3—Li4—O3	58.7 (6)	O2—Li1—O4—Li4	−93.0 (7)
Li1—Li3—Li4—O3	126.2 (7)	O1—Li1—O4—Li4	6.8 (7)
O3—Li3—Li4—O400	73.1 (12)	O100—Li1—O4—Li4	129.1 (8)
O300—Li3—Li4—O400	−30 (2)	Li2—Li1—O4—Li4	−42.2 (7)
O2—Li3—Li4—O400	168.1 (11)	Li3—Li1—O4—Li4	−85.0 (6)
O4—Li3—Li4—O400	−100.4 (13)	O2—Li1—O4—Li3	−7.9 (6)
Li2—Li3—Li4—O400	131.8 (13)	O1—Li1—O4—Li3	91.8 (7)
Li1—Li3—Li4—O400	−160.7 (13)	O100—Li1—O4—Li3	−145.9 (8)
O3—Li3—Li4—Li1	−126.2 (7)	Li2—Li1—O4—Li3	42.9 (6)
O300—Li3—Li4—Li1	130.6 (16)	Li4—Li1—O4—Li3	85.0 (6)
O2—Li3—Li4—Li1	−31.3 (5)	O3—Li3—O4—B4	148.9 (7)
O4—Li3—Li4—Li1	60.3 (6)	O300—Li3—O4—B4	9.5 (12)
Li2—Li3—Li4—Li1	−67.5 (6)	O2—Li3—O4—B4	−114.9 (7)
O3—Li3—Li4—Li2	−58.7 (6)	Li2—Li3—O4—B4	−163.1 (7)
O300—Li3—Li4—Li2	−161.8 (16)	Li4—Li3—O4—B4	153.9 (9)
O2—Li3—Li4—Li2	36.3 (5)	Li1—Li3—O4—B4	−122.4 (8)
O4—Li3—Li4—Li2	127.9 (8)	O3—Li3—O4—Li4	−5.0 (7)
Li1—Li3—Li4—Li2	67.5 (6)	O300—Li3—O4—Li4	−144.4 (9)
O3—Li2—O1—B1	−127.8 (11)	O2—Li3—O4—Li4	91.2 (7)
O2—Li2—O1—B1	136.2 (11)	Li2—Li3—O4—Li4	43.1 (6)
O200—Li2—O1—B1	7.7 (18)	Li1—Li3—O4—Li4	83.7 (6)
Li1—Li2—O1—B1	141.0 (13)	O3—Li3—O4—Li1	−88.7 (6)
Li4—Li2—O1—B1	−135.0 (13)	O300—Li3—O4—Li1	131.9 (9)
Li3—Li2—O1—B1	−174.5 (11)	O2—Li3—O4—Li1	7.5 (6)
O3—Li2—O1—Li1	91.1 (6)	Li2—Li3—O4—Li1	−40.6 (6)
O2—Li2—O1—Li1	−4.8 (5)	Li4—Li3—O4—Li1	−83.7 (6)
O200—Li2—O1—Li1	−133.3 (10)	Li4—O4—B4—C71	−6.8 (18)
Li4—Li2—O1—Li1	84.0 (6)	Li1—O4—B4—C71	121.5 (9)
Li3—Li2—O1—Li1	44.5 (6)	Li3—O4—B4—C71	−137.3 (9)
O3—Li2—O1—Li4	7.1 (6)	Li4—O4—B4—C81	177.1 (10)
O2—Li2—O1—Li4	−88.8 (7)	Li1—O4—B4—C81	−54.6 (12)
O200—Li2—O1—Li4	142.7 (9)	Li3—O4—B4—C81	46.6 (12)
Li1—Li2—O1—Li4	−84.0 (6)	O4—B4—C71—C72	176.2 (8)
Li3—Li2—O1—Li4	−39.5 (7)	C81—B4—C71—C72	−7.4 (11)
O2—Li1—O1—B1	−150.0 (7)	O4—B4—C71—C76	−5.2 (14)
O100—Li1—O1—B1	−15.3 (11)	C81—B4—C71—C76	171.2 (9)
O4—Li1—O1—B1	112.2 (8)	C82—O41—C72—C71	12.5 (14)
Li2—Li1—O1—B1	−155.0 (8)	C82—O41—C72—C73	−168.6 (8)
Li4—Li1—O1—B1	118.9 (8)	C76—C71—C72—O41	178.3 (9)
Li3—Li1—O1—B1	161.3 (7)	B4—C71—C72—O41	−3.0 (13)
O2—Li1—O1—Li2	5.0 (6)	C76—C71—C72—C73	−0.5 (13)
O100—Li1—O1—Li2	139.7 (9)	B4—C71—C72—C73	178.2 (8)
O4—Li1—O1—Li2	−92.8 (7)	O41—C72—C73—C74	−177.5 (9)
Li4—Li1—O1—Li2	−86.2 (6)	C71—C72—C73—C74	1.5 (15)
Li3—Li1—O1—Li2	−43.7 (6)	C72—C73—C74—C75	−2.8 (18)
O2—Li1—O1—Li4	91.2 (7)	C73—C74—C75—C76	3.0 (18)
O100—Li1—O1—Li4	−134.1 (8)	C74—C75—C76—C71	−1.8 (16)
O4—Li1—O1—Li4	−6.7 (7)	C72—C71—C76—C75	0.6 (14)
Li2—Li1—O1—Li4	86.2 (6)	B4—C71—C76—C75	−178.0 (9)
Li3—Li1—O1—Li4	42.4 (6)	O4—B4—C81—C86	4.6 (15)
O4—Li4—O1—B1	−117.7 (7)	C71—B4—C81—C86	−171.8 (9)
O3—Li4—O1—B1	146.6 (7)	O4—B4—C81—C82	−174.3 (8)
O400—Li4—O1—B1	24.4 (12)	C71—B4—C81—C82	9.2 (12)
Li1—Li4—O1—B1	−124.7 (9)	C72—O41—C82—C83	173.7 (10)
Li2—Li4—O1—B1	153.4 (8)	C72—O41—C82—C81	−10.0 (13)
Li3—Li4—O1—B1	−167.4 (7)	C86—C81—C82—C83	−4.1 (16)
O4—Li4—O1—Li2	88.9 (7)	B4—C81—C82—C83	175.0 (10)
O3—Li4—O1—Li2	−6.9 (6)	C86—C81—C82—O41	179.9 (8)
O400—Li4—O1—Li2	−129.1 (9)	B4—C81—C82—O41	−1.0 (13)
Li1—Li4—O1—Li2	81.9 (6)	O41—C82—C83—C84	−179.8 (10)
Li3—Li4—O1—Li2	39.1 (7)	C81—C82—C83—C84	4.0 (18)
O4—Li4—O1—Li1	7.0 (7)	C82—C83—C84—C85	−0.7 (19)
O3—Li4—O1—Li1	−88.7 (7)	C83—C84—C85—C86	−2.1 (17)
O400—Li4—O1—Li1	149.1 (10)	C82—C81—C86—C85	0.9 (15)
Li2—Li4—O1—Li1	−81.9 (6)	B4—C81—C86—C85	−178.1 (10)
Li3—Li4—O1—Li1	−42.8 (7)	C84—C85—C86—C81	1.9 (17)
Li2—O1—B1—C21	4.7 (18)	O2—Li1—O100—C103	−122.4 (10)
Li1—O1—B1—C21	136.4 (9)	O1—Li1—O100—C103	116.2 (9)
Li4—O1—B1—C21	−122.5 (9)	O4—Li1—O100—C103	3.6 (13)
Li2—O1—B1—C11	−175.6 (10)	Li2—Li1—O100—C103	169.2 (10)
Li1—O1—B1—C11	−43.9 (12)	Li4—Li1—O100—C103	62.4 (14)
Li4—O1—B1—C11	57.2 (11)	Li3—Li1—O100—C103	−56.7 (19)
O1—B1—C11—C12	173.5 (8)	O2—Li1—O100—C101	61.7 (11)
C21—B1—C11—C12	−6.8 (11)	O1—Li1—O100—C101	−59.7 (10)
O1—B1—C11—C16	−4.4 (13)	O4—Li1—O100—C101	−172.3 (8)
C21—B1—C11—C16	175.3 (7)	Li2—Li1—O100—C101	−6.7 (15)
C22—O11—C12—C13	−172.5 (8)	Li4—Li1—O100—C101	−113.5 (11)
C22—O11—C12—C11	7.1 (11)	Li3—Li1—O100—C101	127.4 (15)
C16—C11—C12—C13	−1.1 (12)	C103—O100—C101—C102	−61.6 (13)
B1—C11—C12—C13	−179.3 (9)	Li1—O100—C101—C102	114.8 (10)
C16—C11—C12—O11	179.4 (7)	C101—O100—C103—C104	−66.1 (12)
B1—C11—C12—O11	1.2 (11)	Li1—O100—C103—C104	118.1 (11)
O11—C12—C13—C14	180.0 (9)	O1—Li2—O200—C203	76.9 (12)
C11—C12—C13—C14	0.5 (15)	O3—Li2—O200—C203	−157.8 (9)
C12—C13—C14—C15	−0.7 (16)	O2—Li2—O200—C203	−43.0 (12)
C13—C14—C15—C16	1.7 (15)	Li1—Li2—O200—C203	11.9 (15)
C14—C15—C16—C11	−2.6 (14)	Li4—Li2—O200—C203	138.6 (15)
C12—C11—C16—C15	2.2 (12)	Li3—Li2—O200—C203	−99.7 (15)
B1—C11—C16—C15	−179.8 (9)	O1—Li2—O200—C201	−125.3 (9)
O1—B1—C21—C22	−175.3 (8)	O3—Li2—O200—C201	−0.1 (11)
C11—B1—C21—C22	5.0 (12)	O2—Li2—O200—C201	114.8 (9)
O1—B1—C21—C26	6.7 (14)	Li1—Li2—O200—C201	169.6 (9)
C11—B1—C21—C26	−173.0 (8)	Li4—Li2—O200—C201	−63.7 (16)
C12—O11—C22—C23	171.9 (8)	Li3—Li2—O200—C201	58.1 (15)
C12—O11—C22—C21	−9.1 (12)	C203—O200—C201—C202	−81.0 (11)
C26—C21—C22—O11	−179.3 (8)	Li2—O200—C201—C202	118.8 (9)
B1—C21—C22—O11	2.5 (13)	C201—O200—C203—C204	173.7 (10)
C26—C21—C22—C23	−0.4 (13)	Li2—O200—C203—C204	−26.9 (14)
B1—C21—C22—C23	−178.7 (9)	O3—Li3—O300—C301	−82.5 (14)
O11—C22—C23—C24	179.5 (9)	O2—Li3—O300—C301	150.6 (12)
C21—C22—C23—C24	0.6 (15)	O4—Li3—O300—C301	40.6 (15)
C22—C23—C24—C25	−1.1 (16)	Li2—Li3—O300—C301	−154.9 (17)
C23—C24—C25—C26	1.4 (16)	Li4—Li3—O300—C301	−9(2)
C24—C25—C26—C21	−1.3 (15)	Li1—Li3—O300—C301	96.4 (14)
C22—C21—C26—C25	0.8 (14)	O3—Li3—O300—C303	118.8 (10)
B1—C21—C26—C25	179.0 (10)	O2—Li3—O300—C303	−8.1 (12)
O1—Li1—O2—B2	122.7 (11)	O4—Li3—O300—C303	−118.1 (9)
O100—Li1—O2—B2	−5.2 (17)	Li2—Li3—O300—C303	46 (2)
O4—Li1—O2—B2	−140.9 (11)	Li4—Li3—O300—C303	−167.4 (13)
Li2—Li1—O2—B2	127.6 (12)	Li1—Li3—O300—C303	−62.3 (13)
Li4—Li1—O2—B2	171.6 (11)	C303—O300—C301—C302	−174.2 (11)
Li3—Li1—O2—B2	−148.9 (13)	Li3—O300—C301—C302	24.9 (19)
O1—Li1—O2—Li2	−4.9 (6)	C301—O300—C303—C304	79.8 (15)
O100—Li1—O2—Li2	−132.8 (9)	Li3—O300—C303—C304	−118.1 (12)
O4—Li1—O2—Li2	91.5 (6)	O4—Li4—O400—C403	125.5 (9)
Li4—Li1—O2—Li2	44.0 (5)	O1—Li4—O400—C403	−5.3 (13)
Li3—Li1—O2—Li2	83.5 (6)	O3—Li4—O400—C403	−117.5 (8)
O1—Li1—O2—Li3	−88.4 (6)	Li1—Li4—O400—C403	54.2 (19)
O100—Li1—O2—Li3	143.7 (9)	Li2—Li4—O400—C403	−64.6 (13)
O4—Li1—O2—Li3	8.0 (6)	Li3—Li4—O400—C403	−166.3 (10)
Li2—Li1—O2—Li3	−83.5 (6)	O4—Li4—O400—C401	−73.3 (13)
Li4—Li1—O2—Li3	−39.5 (6)	O1—Li4—O400—C401	155.8 (9)
O1—Li2—O2—B2	−144.7 (7)	O3—Li4—O400—C401	43.7 (11)
O3—Li2—O2—B2	117.2 (7)	Li1—Li4—O400—C401	−144.7 (14)
O200—Li2—O2—B2	−12.0 (11)	Li2—Li4—O400—C401	96.6 (12)
Li1—Li2—O2—B2	−149.6 (8)	Li3—Li4—O400—C401	−5.2 (16)
Li4—Li2—O2—B2	167.2 (7)	C403—O400—C401—C402	−171.2 (10)
Li3—Li2—O2—B2	123.4 (8)	Li4—O400—C401—C402	26.1 (14)
O1—Li2—O2—Li1	4.9 (6)	C401—O400—C403—C404	79.8 (11)
O3—Li2—O2—Li1	−93.1 (6)	Li4—O400—C403—C404	−117.0 (10)
